# Band Gap Characters and Ferromagnetic/Antiferromagnetic Coupling in Group-IV Monolayers Tuned by Chemical Species and Hydrogen Adsorption Configurations

**DOI:** 10.1186/s11671-015-1040-y

**Published:** 2015-09-04

**Authors:** Wen-Zhe Yu, Jia-An Yan, Shang-Peng Gao

**Affiliations:** Department of Materials Science, Fudan University, Shanghai, 200433 China; Department of Physics, Astronomy, and Geosciences, Towson University, Towson, MD 21252 USA

**Keywords:** Group-IV monolayers, Semihydrogenation, Band gap, FM/AFM coupling, DFT

## Abstract

One-side semihydrogenated monolayers of carbon, silicon, germanium, and their binary compounds with different configurations of hydrogen atoms are investigated by density functional theory. Among three considered configurations, zigzag, other than the most studied chair configuration, is energetically the most favorable structure of one-side semihydrogenation. Upon semihydrogenation, the semimetallic silicene, germanene, and SiGe become semiconductors, while the band gap in semiconducting SiC and GeC is reduced. Semihydrogenated silicene, germanene, SiGe, and GeC with chair configuration are found to be ferromagnetic semiconductors. For semihydrogenated SiC, it is ferromagnetic when all hydrogen atoms bond with silicon atoms, while an antiferromagnetic coupling is predicted when all hydrogen atoms bond with carbon atoms. The effect of interatomic distance between two neighboring magnetic atoms to the ferromagnetic or antiferromagnetic coupling is studied. For comparison, properties of one-side and both-side fully hydrogenated group-IV monolayers are also calculated. All fully hydrogenated group-IV monolayers are nonmagnetic semiconductors with band gaps larger than those of their semihydrogenated counterparts.

**Electronic supplementary material:**

The online version of this article (doi:10.1186/s11671-015-1040-y) contains supplementary material, which is available to authorized users.

## Background

Currently, tremendous attention has been focused on two-dimensional (2D) monolayers composed of group-IV elements, such as carbon, silicon, and germanium. Theoretical and experimental studies show that silicon [1–16] and germanium [4, 17–21] can form graphene-like structures, namely silicene and germanene. The first hypothesis of silicene and germanene can be traced back to 1994 [22], but the experimental synthesis becomes possible only in recent years. Fabrications of silicene on various substrates such as silver [6–13], iridium [14], and zirconium diboride [15] and germanene on gold [19], platinum [20], and gallium arsenide [21] have been reported. Different from graphene that prefers a perfectly planar structure, silicene and germanene are stabilized by a low-buckled structure. They both, however, resemble the electronic characteristic of graphene, i.e., an energy band crossing at the Dirac cone and linear dispersion around the crossing [4, 5, 13, 23–26]. By applying an external electric field perpendicular to the silicene or germanene lattices [27–32], the band gap of silicene and germanene can be opened. The opening of the band gap as well as introducing the quantum anomalous Hall effect in silicene or germanene can also be obtained by decorating 3d or 5d transition metal atoms [33–36] or small organic molecule adsorption [37]. In addition to silicene and germanene which consist only one type of element, monolayer compounds consisting two types of elements are also of great interest, including planar silicon carbide (SiC) [26, 38–43] and germanium carbide (GeC) [26, 43] which open a band gap to become semiconducting, and low-buckled silicon germanide (SiGe) [26, 44, 45] with a band structure similar to those of graphene and silicene.

With good feasibility, reversibility, and controllability [46, 47], hydrogenation is a promising method to further tune the properties of 2D monolayers and to expand the scope of their application. Previous work found that different ratio of hydrogenation significantly changes the electronic and magnetic properties of group-IV monolayers [44, 45, 48–62]. Full hydrogenation opens a band gap for silicene and germanene [53–59] and enlarges the band gap of monolayer SiC [44, 59–61]. Among boat, chair, and zigzag configurations of fully hydrogenated silicene [53, 56, 58], germanene [53], and monolayer SiC [60, 61], chair configuration has the lowest energy. After full hydrogenation, hydrogen atoms on one side can be removed to achieve one-side semihydrogenation on the other side. Based on phonon calculation, Wang et al. confirmed the dynamical stability of semihydrogenated silicene and germanene with chair configuration and they reported that both silicene and germanene become ferromagnetic semiconductors upon semihydrogenation with chair configuration [49]. Ferromagnetism in semihydrogenated silicene with chair configuration was predicted using first principles by other groups as well [50, 56, 57]. In monolayer SiC, ferromagnetism or antiferromagnetism can be induced by semihydrogenation with chair configuration when hydrogen atoms bond with silicon atoms or carbon atoms, respectively, and this kind of hydrogenation slightly reduces the band gap of monolayer SiC [44, 48]. Zhou et al. stated that semihydrogenated monolayer SiGe with chair configuration is a ferromagnetic semiconductor, no matter hydrogen atoms bond with silicon or germanium atoms [45]. Ma et al. found ferromagnetism in semihydrogenated monolayer GeC with chair configuration [62].

Up to now, most studies of semihydrogenated group-IV monolayers are limited to the chair configuration [44, 45, 48–50, 56, 57]. One-side semihydrogenation with boat and zigzag configurations is only examined in silicene [56]. The energetic, electronic, and magnetic properties of semihydrogenated germanene, SiC, GeC, and SiGe with boat and zigzag configurations still remain unclear. There is, however, neither theoretical nor experimental evidence can prove the preference of chair configuration to boat, zigzag, and other configurations. In this work, based on our first-principle calculations on the total energies of one-side semihydrogenated silicene, germanene, SiC, GeC, and SiGe monolayers with boat, chair, and zigzag configurations, zigzag configuration is shown to have the lowest energy for all considered group-IV monolayers. The effect of semihydrogenation to the electronic properties of semihydrogenated group-IV monolayers are revealed by band structure calculations. For silicene, germanene, and monolayer SiGe, semihydrogenation opens a band gap, while for monolayer SiC and GeC, the semiconducting band gap is reduced by semihydrogenation. Ferromagnetism or antiferromagnetism exists in semihydrogenated group-IV monolayers only with chair configuration. The distance between two neighboring magnetic atoms is a factor that influences whether ferromagnetic or antiferromagnetic coupling forms. If further hydrogenated to full hydrogenation, all semihydrogenated group-IV monolayers become nonmagnetic semiconductors with larger band gaps.

## Calculation Method

All calculations are performed using the pseudopotential plane-wave method as implemented in the CASTEP code [63]. The generalized gradient approximation (GGA) with the Perdew-Burke-Ernzerhof (PBE) [64] parametrization and norm-conserving pseudopotential are used for most calculations unless mentioned otherwise. The cut-off energy for the plane-wave basis set is 1000 eV. To carry out the Brillouin-zone integration, 12 × 12 × 1 Monkhorst-Pack k-point grid [65] is used for one-side semihydrogenated silicene, germanene, SiGe, SiC, and GeC monolayers with chair configuration and 6 × 12 × 1 k-point grid for boat and zigzag configurations. The unit cell of semihydrogenated group-IV monolayers with boat or zigzag configuration is twice as large as the unit cell of chair configuration. To avoid spurious interactions between adjacent layers, a vacuum space of 20 Å is introduced along the direction perpendicular to the 2D plane. Semi-empirical dispersion interaction correction [66] with TS method [67] is adopted to treat the van der Waals interaction of the layered system. The lattice parameters and atomic positions are fully relaxed under Broyden-Fletceher-Goldfarb-Shanno (BFGS) [68] scheme until the energy difference between two steps is smaller than 5 × 10^−6^ eV/atom, and the maximum force is smaller than 0.01 eV/Å. All the computational parameters mentioned above are carefully tested to achieve convergence of the total energy. There is no spin-orbit coupling implemented in the CASTEP code.

The formation energies of atomic hydrogen on the group-IV monolayers are defined as

$$ {E}_f=\left({E}_{\mathrm{tot}}-{E}_0-{N}_H{E}_H\right)/{N}_H $$

where *E*_tot_ and *E*_0_ are the total energy of the hydrogenated and unhydrogenated group-IV monolayers, respectively; *N*_*H*_ is the number of adsorbed hydrogen atoms; and *E*_*H*_ is the total energy of isolated hydrogen atom. This definition of formation energy can characterize the hydrogenation process of group-IV monolayers.

## Results and Discussion

### Structure and Stability

**Fig. 1:**
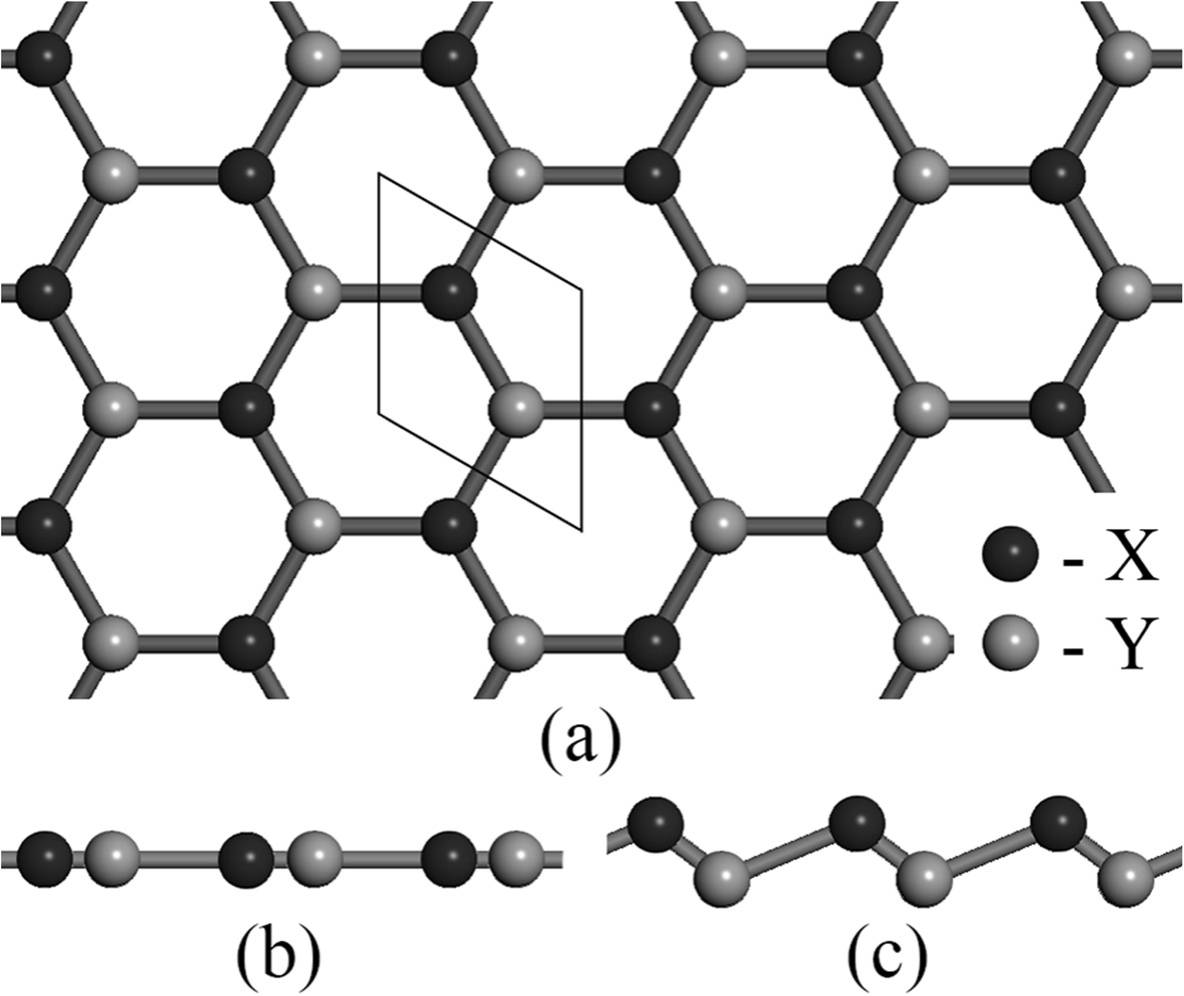
**a** *Top view* of the hexagonal structure of monolayer silicene (X = Y = Si), germanene (X = Y = Ge), silicon germanide (X = Si, Y = Ge), silicon carbide (X = Si, Y = C), and germanium carbide (X = Ge, Y = C). Monolayer SiC and GeC prefer a planar structure (**b**), while silicene, germanene, and monolayer SiGe prefer a low-buckled structure (**c**)

**Fig. 2:**
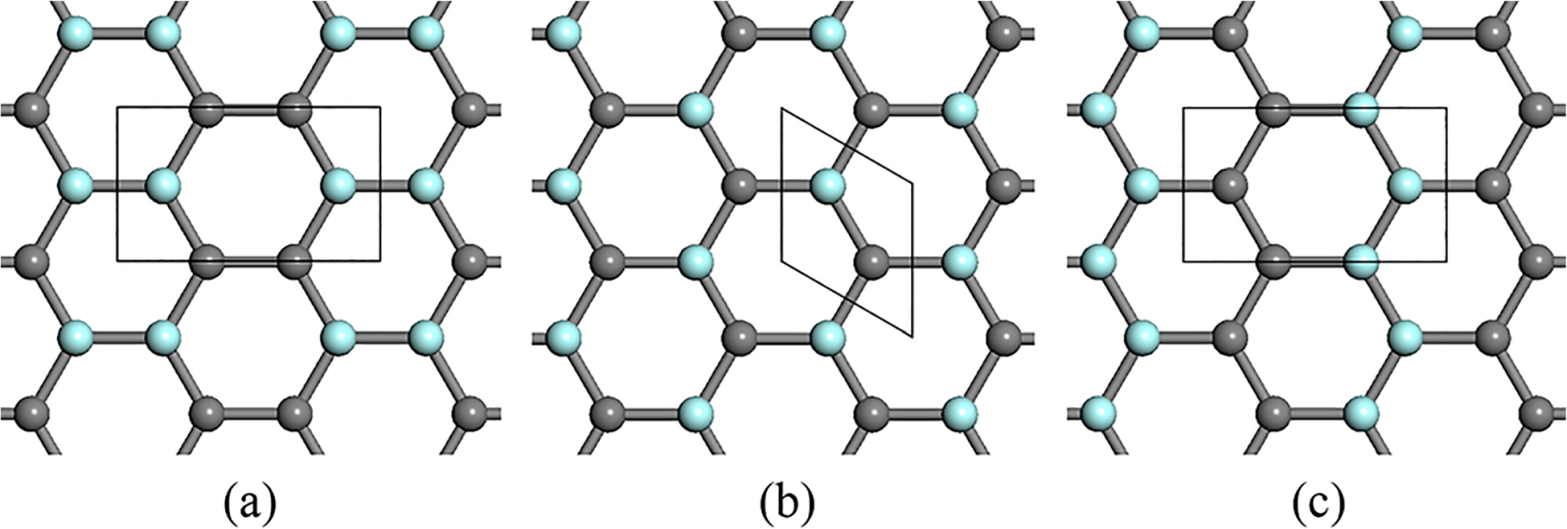
Schematics of the boat (**a**), chair (**b**), and zigzag (**c**) configurations of one-side semihydrogenation. Hydrogen atoms are on *top* of the 2D plane and represented by the *blue spheres*. Unit cell of each structure is marked by the *black box*

Top and side view of group-IV monolayers are depicted in Fig. 1. Without hydrogenation, graphene holds a strictly planar structure due to the sp^2^ hybridization of carbon atom. Unhydrogenated monolayer SiC and GeC are also stable as planar structures (Fig. 1b). On the contrary, silicon and germanium atoms tend to be sp^3^ hybridized, thus leading to low-buckled structures of silicene, germanene, and monolayer SiGe (Fig. 1c). In our calculations, we consider three configurations of one-side semihydrogenation on group-IV monolayers, i.e., boat, chair, and zigzag (Fig. 2).

The buckling constants as well as lattice constants of semihydrogenated group-IV monolayers are summarized in Table 1. After semihydrogenated, SiC and GeC monolayers become buckled. Buckling constants of silicene, germanene, and monolayer SiGe increase upon semihydrogenation. Group-IV atom bonded with a hydrogen atom forms four σ bonds with three group-IV atoms and one hydrogen atom as nearest neighbors, similar to sp^3^ hybridization. Lattice constants of semihydrogenated group-IV monolayers are slightly larger than those of their unhydrogenated counterparts. For example, lattice constant of silicene is 3.876 Å, smaller than lattice constants of semihydrogenated silicene with boat, chair, and zigzag configurations, which are 3.897, 3.897, and 3.880 Å, respectively. According to previous reports [49, 50, 53–56, 58–61], the enlargement of lattice constant by semihydrogenation or full hydrogenation is common in group-IV monolayer systems. There is a discrepancy no larger than 2 % between our calculated lattice constants and those in other reports using DFT [49, 50, 56], which is common in literatures due to the different computational parameters, especially the exchange-correlation functional and pseudopotential dataset. In semihydrogenated group-IV monolayers with chair configuration, each hydrogen atom sits directly above the corresponding group-IV atom it bonds with, while in the cases of boat and zigzag configurations, hydrogen atoms are not strictly above the corresponding group-IV atoms. Detailed information regarding the lattice parameters and atomic positions are provided in Additional file 1.

**Table 1:** Lattice constants, buckling constants, formation energies (*E*_*f*_), band gap energies (*E*_*g*_), and magnetism of one-side semihydrogenated silicene, germanene, SiGe, SiC, and GeC monolayers with boat, chair, and zigzag configurations. The NM, FM, and AFM represent nonmagnetism, ferromagnetism, and antiferromagnetism, respectively

	Lattice constant (Å)	Buckling constant (Å)	*E*_*f*_ (eV)	*E*_*g*_ (eV)	Magnetism
Silicene	3.876	0.444	-	Metal	NM
Chair	3.897	0.670	−3.272	1.094	FM
	3.895(e)	0.676(e)		0.94(e)	FM(e, g, h, i)
	3.861(f)	0.691(f)		1.74(f)	
	3.85(g)			0.95(g)	
	3.896(h)			0.84(h)	
				1.79(i)	
Boat	3.897	1.282	−3.579	1.105	NM
	3.898(h)			0.84(h)	NM(h, i)
				1.79(i)	
Zigzag	3.880	1.226	−3.612	0.161	NM
	3.878(h)			Metal(h)	NM(h, i)
				Metal(i)	
Germanene	4.030	0.683	-	Metal	NM
Chair	4.076	0.751	−2.975	0.652	FM
	4.111(e)	0.758(e)		0.41(e)	FM(e)
	4.057(f)	0.742(f)		1.32(f)	
Boat	4.094	1.321	−3.360	0.262	NM
Zigzag	4.164	1.266	−3.373	0.347	NM
SiGe	3.963	0.573	-	Metal	NM
Chair (H-SiGe)	4.003	0.704	−3.295	0.944	FM
		0.7418(b)		0.6485(b)	FM(b)
				1.495(c)	
Chair (SiGe-H)	4.003	0.702	−2.985	1.099	FM
		0.7436(b)		0.8604(b)	FM(b)
Boat	4.008	1.069	−3.424	1.133	NM
Zigzag	4.046	1.151	−3.457	0.243	NM
SiC	3.083	0	-	2.586	NM
Chair (H-SiC)	3.141	0.378	−2.496	0.940	FM
		0.32(a)		0.81(a)	FM(a, d)
		0.378(d)		0.85(d)	
Chair (SiC-H)	3.100	0.583	−2.866	1.469	AFM
		0.62(a)		0.34(a)	FM(a)
		0.572(d)		1.19(d)	AFM(d)
Boat	3.150	0.520	−3.339	2.120	NM
Zigzag	3.170	0.696	−3.440	1.577	NM
GeC	3.231	0	-	2.183	NM
Chair (H-GeC)	3.288	0.422	−2.511	1.234	FM
Chair (GeC-H)	3.268	0.618	−3.313	1.007	FM
Boat	3.300	0.586	−3.430	1.661	NM
Zigzag	3.332	0.725	−3.475	1.222	NM

LDA: a, [44]. GGA: b, [45]; d, [48]; e, [49]; g, [50]; h, [56]. HSE06: c, [45]; f, [49]; i, [55]

Depending on which type of elements hydrogen atoms bond with, there are two possible structures for semihydrogenated monolayer SiGe with chair configuration. The structure in which all hydrogen atoms bond with silicon atoms is denoted by H-SiGe, and the structure in which all hydrogen atoms bond with germanium atoms is denoted by SiGe-H. For semihydrogenated monolayer SiC and GeC with chair configuration, the same denotation is used. In each case of semihydrogenated monolayer SiGe, SiC, and GeC with chair configuration, formation energies are different when hydrogen atoms are bonded with different group-IV atoms, indicating a bonding preference of hydrogen to different group-IV elements. Formation energies of H-SiGe, SiC-H, and GeC-H are lower than those of SiGe-H, H-SiC, and H-GeC, respectively. Thus, hydrogen atoms have higher bonding preference to silicon atoms than to germanium atoms in monolayer SiGe and have higher preference to carbon atoms than to silicon (germanium) atoms in monolayer SiC (GeC), i.e., hydrogen atoms prefer to bond with the lighter element in the monolayer group-IV binary compound. For monolayer SiGe, Zhou et al. found the same bonding preference as us, and they believed this bonding preference facilitates the synthesis of one-side semihydrogenated SiGe with chair configuration [45]. Indeed, due to the different types of elements in the unit cells of monolayer SiGe, SiC, and GeC, it seems that when hydrogenation process occurs, hydrogen atoms will tend to bond with one particular type of atoms to form chair configuration. Our calculation, however, gives an energetic order of zigzag < boat < chair, in semihydrogenated silicene, germanene, as well as binary SiGe, SiC, and GeC monolayers. Formation energy of boat configuration is slightly higher than that of zigzag configuration, while formation energy of chair configuration is much higher than those of boat and zigzag configurations (see Table 1). Exchange-correlation functional of local density approximation (LDA) and ultrasoft pseudopotential are employed to recalculate the energies of semihydrogenated silicene and monolayer SiC for comparison. A same order of energies is confirmed. In fact, this order has also been found for semihydrogenated graphene and semifluorinated graphene in our previous DFT calculation [69]. Therefore, zigzag configuration, other than the most studied chair configuration, has the lowest formation energy among different configurations of one-side semihydrogenated group-IV monolayers. If directly hydrogenating one side of group-IV monolayers, zigzag configuration is more likely to appear than chair and boat configurations.

It has been reported that for fully hydrogenated silicene [53, 56, 58], germanene [53], and monolayer SiC [60, 61], chair configuration has lower energy than boat and zigzag configurations. Our calculations confirmed these reported results and showed that similar conclusion can be applied to monolayer SiGe and GeC. We calculated the formation energies of atomic hydrogen on the monolayers for fully hydrogenated silicene, germanene, SiGe, SiC, and GeC. According to our results, formation energies of both-side full hydrogenation are lower than those of one-side full hydrogenation. Chair configuration has the lowest formation energy among both-side fully hydrogenated group-IV monolayers with boat, chair, and zigzag configurations (See Table 2. Detailed information regarding the structures of the fully hydrogenated group-IV monolayers can be found in Additional file 1). Therefore, we propose a two-step strategy to achieve semihydrogenation with chair configuration in group-IV monolayers: first, fully hydrogenate the group-IV monolayers to get chair configuration on both side; second, remove hydrogen atoms from one side to get chair configuration of semihydrogenation on the other side.

**Table 2:** Formation energies (*E*_*f*_) and band gap energies (*E*_*g*_) of one-side and both-side fully hydrogenated silicene, germanene, SiGe, SiC, and GeC monolayers with chair, boat, and zigzag configurations

	Configuration	*E*_*f*_ (eV)	*E*_*g*_ (eV)
Silicene	One-side	−5.935	1.893
	Chair	−7.005	2.207
	Boat	−6.962	1.753
	Zigzag	−6.966	2.293
Germanene	One-side	−5.345	1.709
	Chair	−6.312	1.327
	Boat	−6.265	1.652
	Zigzag	−6.270	2.118
SiGe	One-side	−5.182	1.826
	Chair	−6.195	1.915
	Boat	−6.157	1.690
	Zigzag	−6.160	2.247
SiC	One-side	−6.055	3.575
	Chair	−7.062	3.953
	Boat	−7.024	4.298
	Zigzag	−7.028	3.905
GeC	One-side	−5.165	3.180
	Chair	−6.262	3.528
	Boat	−6.220	3.619
	Zigzag	−6.225	3.935

### Electronic Property

Band structure calculation is carried out for semihydrogenated group-IV monolayers with boat, chair, and zigzag configurations. According to our analysis of the energetic stability, chair configuration is not energetically the most favorable among the three considered configurations of one-side semihydrogenation. We present, for the first time, the band structures of one-side semihydrogenated germanene, SiGe, SiC, and GeC monolayers with boat and zigzag configurations.

**Fig. 3:**
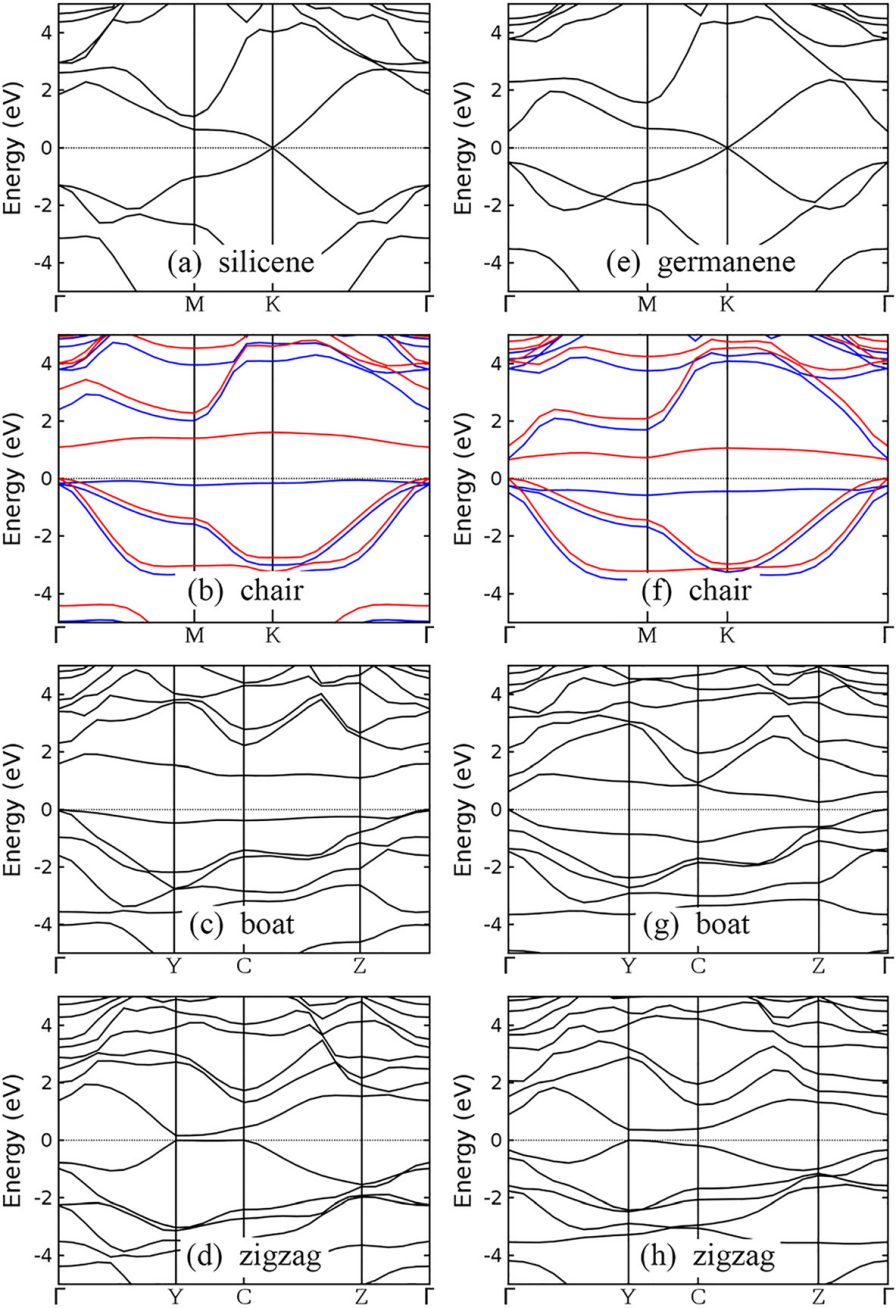
Band structures of silicene (**a**), germanene (**e**), and their semihydrogenated counterparts with chair, boat, and zigzag configurations (panels **b–d** are for semihydrogenated silicene and panels **f–h** are for semihydrogenated germanene). The Fermi level or the valence band maximum has been set to 0 eV and indicated by the *black dotted line*. For chair configuration, majority spin and minority spin bands are indicated by *blue* and *red lines*, respectively

Band structures of silicene, germanene, and their semihydrogenated counterparts with chair, boat, and zigzag configurations are illustrated in Fig. 3. Although the low-buckled structures of silicene and germanene are different from the planar structure of graphene, the band structures of silicene and germanene still keep the characteristic of that of graphene. Thanks to the preserved symmetry between two sublattices, the linear dispersion around Dirac cone exists in both silicene and germanene (Fig. 3a, e). Note that previous publications have reported that the inclusion of spin-orbit coupling can open band gaps for silicene and germanene [31, 32, 66, 70]. Typical reported values of the spin-orbit coupling energies are about 1.55 meV for silicene and 23.9 meV for germanene [70]. The small band gap opening induced by spin-orbit coupling is crucial for properties such as quantum spin Hall effect in silicene or germanene [70]. But for the band gaps of semihydrogenation group-IV monolayers at the order of 1 eV discussed below, neglecting the spin-orbit coupling is still a good approximation to calculate the band gap energy.

**Fig. 4:**
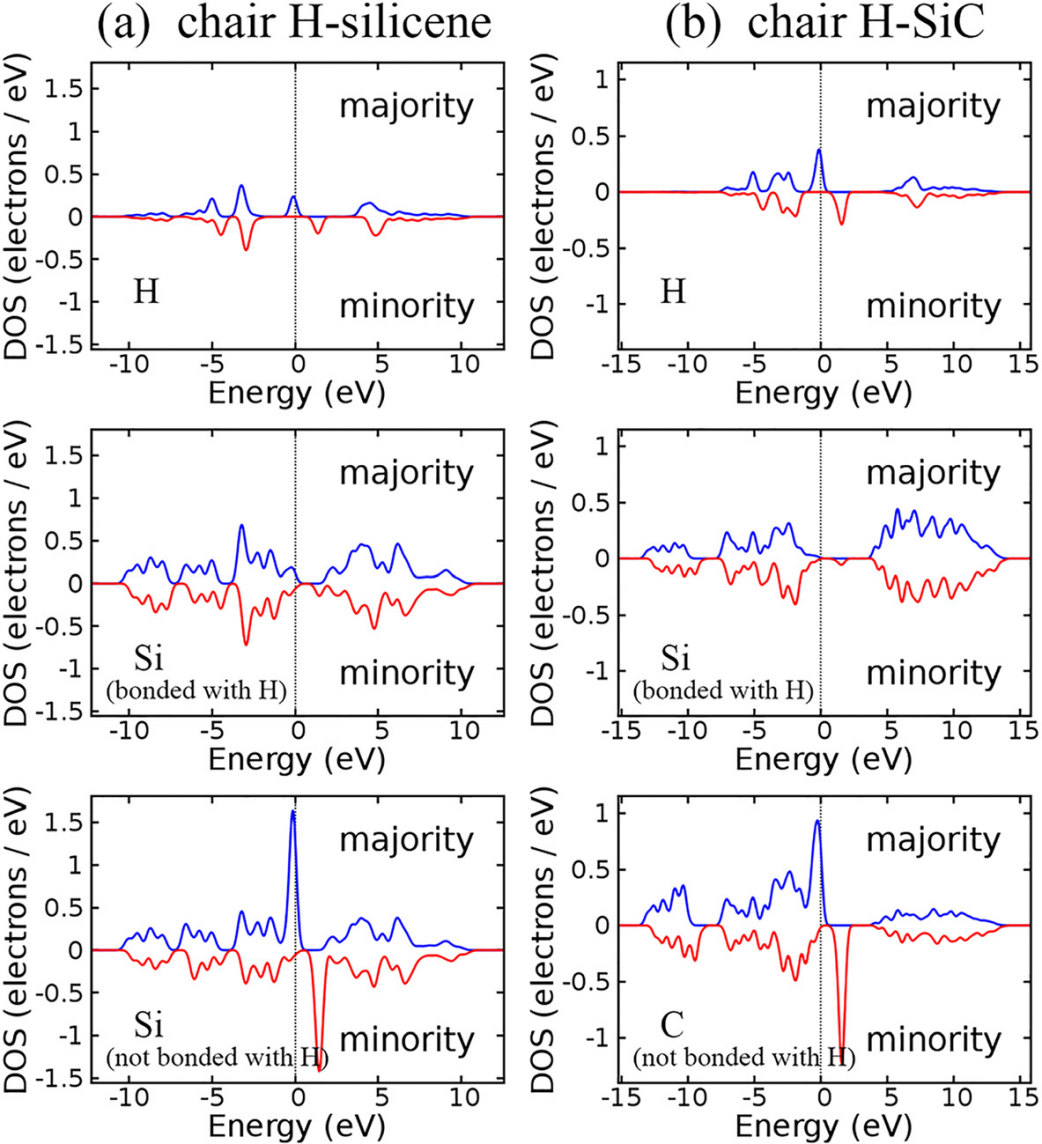
Density of states (DOS) located at each atom in semihydrogenated silicene with chair configuration (**a**) and semihydrogenated monolayer SiC with chair H-SiC configuration (**b**)

After one-side semihydrogenation, the H-Si (H-Ge) bond makes silicene (germanene) semiconducting, independent of the arrangement of hydrogen atoms. Semihydrogenated silicene and germanene with chair configurations are magnetic semiconductors with band gaps of 1.094 and 0.652 eV, respectively (Fig. 3b, f). The two flat bands around the band gaps correspond to the group-IV atoms that do not bond with hydrogen atoms, as shown by the density of states (DOS) plotted in Fig. 4. Similar DOS of atoms in semihydrogenated germanene, SiGe, and GeC monolayers with chair configuration is given in Additional file 1 for completeness.

Semihydrogenated silicene and germanene with boat and zigzag configurations are semiconducting. Indirect band gaps of 1.105 and 0.262 eV are predicted for semihydrogenated silicene and germanene with boat configuration, respectively (Fig. 3c, g), while direct band gaps of 0.161 and 0.347 eV are predicted for semihydrogenated silicene and germanene with zigzag configuration, respectively (Fig. 3d, h). A metallic nature was reported by Zhang et al. for semihydrogenated silicene with zigzag configuration [56], inconsistent with what we found. To confirm our result, LDA and ultrasoft pseudopotential are used to recalculate the electronic structure of semihydrogenated silicene with zigzag configuration and a band dispersion similar to that calculated using GGA and norm-conserving pseudopotential is obtained, with a slightly smaller band gap of 0.132 eV.

Our calculated band gap energies of semihydrogenated silicene and germanene are close to other reported DFT-GGA results [49, 50, 56]. It is well known that DFT calculation at LDA or GGA level underestimates band gap energy. Using HSE06 functional and taking spin-orbit coupling into consideration, Wang et al. calculated band gaps of semihydrogenated silicene and germanene with chair configuration and reported values of 1.74 and 1.32 eV, respectively [49]. Also using HSE06 functional, Zhang et al. stated that band gap energies for semihydrogenated silicene with chair and boat configurations are both 1.79 eV [56]. These reported HSE06 band gap energies are larger than our DFT-GGA results. In addition to hydrogenation, decorating silicene and germanene with transition metal atoms or small organic molecules also opens a band gap and simultaneously induces a quantum anomalous Hall state [33–37]. By applying an external electric field perpendicular to the 2D plane, a band gap increasing with the electric field strength is obtained in silicene and germanene [27, 28, 30, 31].

**Fig. 5:**
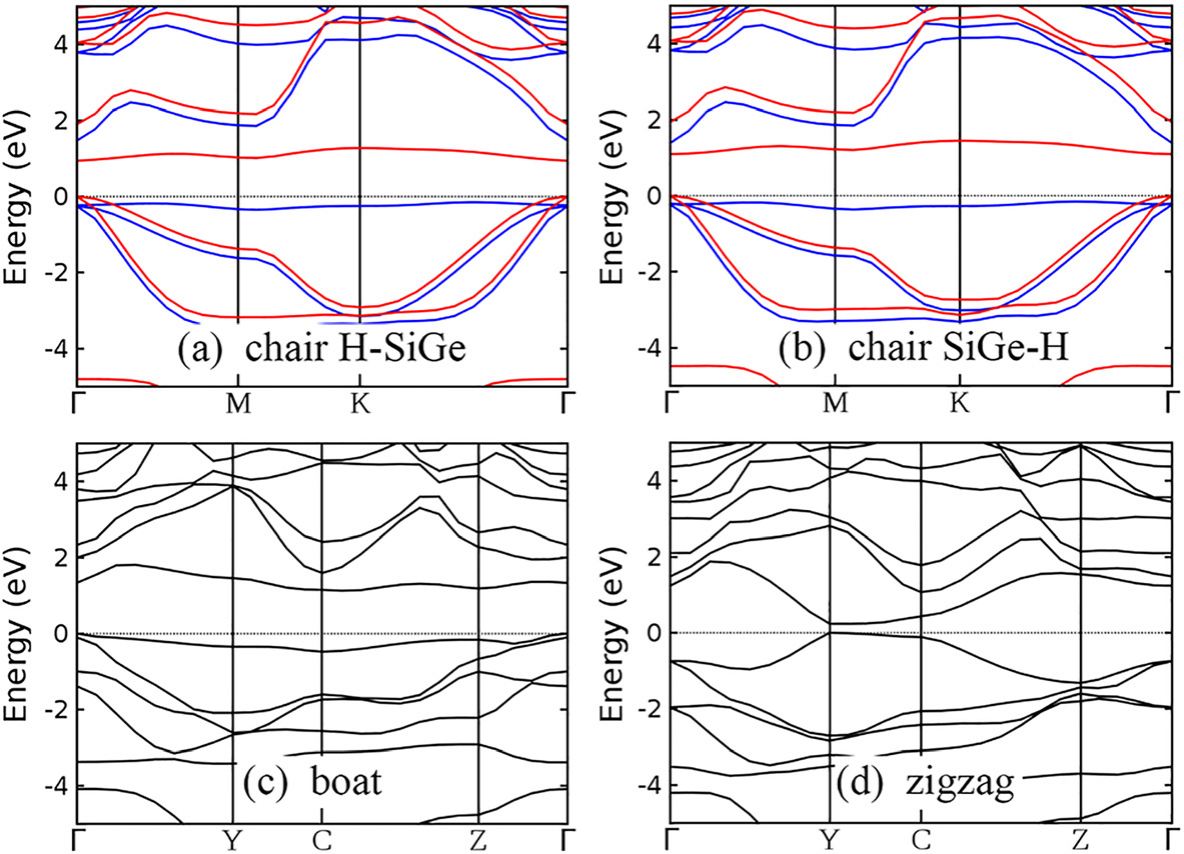
Band structures of semihydrogenated monolayer SiGe with chair H-SiGe (**a**), chair SiGe-H (**b**), boat (**c**), and zigzag (**d**) configurations. The valence band maximum has been set to 0 eV and indicated by the *black dotted line*. For chair configuration, majority spin and minority spin bands are indicated by *blue* and *red lines*, respectively

The band structures of monolayer SiGe, silicene, and germanene and the effect of semihydrogenation to their band structures are similar, which is attributed to the similarity between silicon and germanium atoms. Both H-SiGe and SiGe-H are magnetic semiconductors with band gaps of 0.944 and 1.099 eV, respectively (Fig. 5a, b). Semihydrogenation of boat configuration opens an indirect band gap of 1.133 eV for monolayer SiGe (Fig. 5c), while semihydrogenation of zigzag configuration opens a small direct band gap of 0.243 eV (Fig. 5d).

**Fig. 6:**
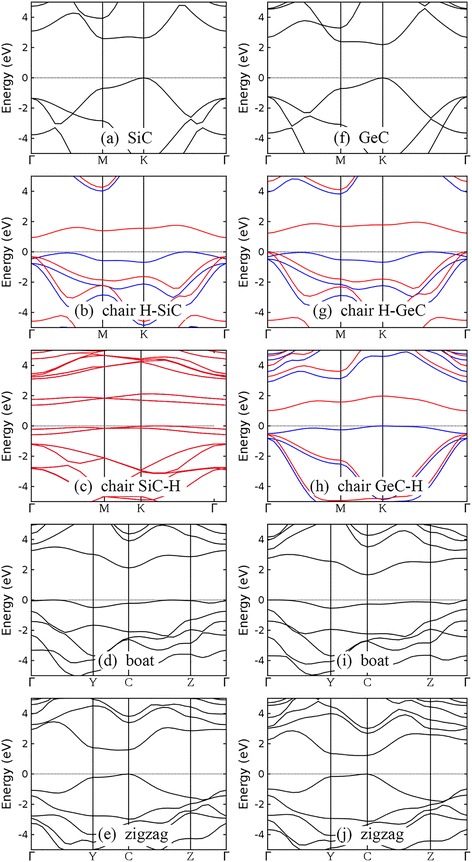
Band structures of monolayer SiC (**a**), GeC (**f**), and their semihydrogenated counterparts with chair, boat, and zigzag configurations (panels **b–e** are for semihydrogenated SiC and panels **g–j** are for semihydrogenated GeC). The valence band maximum has been set to 0 eV and indicated by the *black dotted line*. For chair configuration, majority spin and minority spin bands are indicated by *blue* and *red lines*, respectively

In spite of the graphene-like planar structures of monolayer SiC and GeC, the inversion symmetry between two sublattices is broken, leading to semiconducting behaviors. Our DFT-GGA results show that monolayer SiC has an indirect band gap of 2.586 eV, while monolayer GeC has a direct band gap of 2.183 eV (Fig. 6a, f). Note that no agreement has been reached on the type of band gap of monolayer SiC. Both direct [39, 40, 43] and indirect [26, 38–40, 43] band gaps are reported. According to our results, the energies of the lowest valence band at M point and K point are very close. The valence band minimum of monolayer SiC is located at M, while energy at K is only 0.04 eV higher. This kind of small band dispersion variation is difficult to identify experimentally and is sensitive to the pseudopotentials employed in the calculation [71]. One-side semihydrogenation reduces the band gap of monolayer SiC and GeC, regardless of the configuration of hydrogen atoms. All chair-like structures, i.e., H-SiC, SiC-H, H-GeC, and GeC-H, are magnetic semiconductors with band gaps smaller than their unhydrogenated counterparts (Fig. 6b, c, g, h). Band gaps of monolayer SiC and GeC decrease after semihydrogenation with boat and zigzag configurations. Semihydrogenation of boat configuration brings indirect band gaps (Fig. 6d, i), while semihydrogenation of zigzag configuration opens direct band gaps (Fig. 6e, j).

One-side and both-side fully hydrogenated group-IV monolayers with boat, chair, and zigzag configurations are all semiconductors with band gaps considerably larger than those of their semihydrogenated counterparts. Their band gap energies are listed in Table 2. All both-side fully hydrogenated group-IV monolayers except for fully hydrogenated silicene with chair configuration and fully hydrogenated germanene with zigzag configuration have direct band gaps, while all one-side fully hydrogenated group-IV monolayers as well as fully hydrogenated silicene with chair configuration and fully hydrogenated germanene with zigzag configuration have indirect band gaps. Detailed band structures of fully hydrogenated group-IV monolayers are provided in Additional file 1.

### Magnetic Property

Our spin-polarized calculation shows that one-side semihydrogenated group-IV monolayers with boat and zigzag configurations are non-spin polarized, while semihydrogenated group-IV monolayers with chair configuration are spin polarized. As discussed in “Structure and Stability” section, since zigzag configuration of semihydrogenation is energetically more preferable than boat and chair configurations, it is more probable to obtain nonmagnetic semiconductor with zigzag configuration by direct one-side semihydrogenation to group-IV monolayers. However, when it comes to full hydrogenation, chair configuration has the lowest total energy [53, 56, 58, 60, 61]. Therefore, magnetism in group-IV monolayers must be achieved through the two-step strategy: first fully hydrogenating to get chair configuration on both side, then removing hydrogen atoms from one side to get semihydrogenation with chair configuration on the other side.

**Fig. 7:**
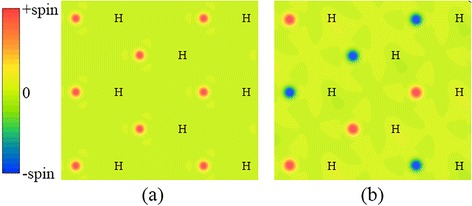
Spin density of semihydrogenated monolayer SiC with chair configuration. H-SiC structure (**a**) is ferromagnetic, while SiC-H structure (**b**) is antiferromagnetic

For chair configuration, there are two types of group-IV atoms, classified either by the different types of atoms (in monolayer SiGe, SiC, and GeC) or by the different chemical environments of atoms (in silicene and germanene). These two types of atoms are hereafter referred to as IV_1_ and IV_2_. Before hydrogenation, weak π bonds exist between IV_1_ and IV_2_ atoms. When IV_1_ atoms bond with hydrogen atoms, strong σ bonds will form between IV_1_ and hydrogen. The network of π bonds is destroyed, leaving p_z_ electrons in IV_2_ atoms localized and unpaired. As shown in Fig. 7, spin density is more significant at the IV_2_ atoms that are not bonded with hydrogen atoms, no matter hydrogen atoms bond with silicon or carbon atoms. In other words, the spin moments are mainly carried by the IV_2_ atoms, whereas there are only very small spin moments carried by IV_1_ and hydrogen atoms. For the case of single hydrogen atom adsorbed on graphene, Sofo et al. have shown that the spin polarization is small in the carbon atom directly bonded with the hydrogen atom and the magnetic moment is larger at its neighboring carbon atoms [72]. Spin density of other ferromagnetic structures shows similar pattern as the H-SiC structure, thus is not presented here. For fully hydrogenated group-IV monolayers, our calculations show that the magnetism disappears, which can be attributed to the lack of unpaired electrons.

Total energies of nonmagnetic (NM), ferromagnetic (FM), and antiferromagnetic (AFM) states are compared for each semihydrogenated group-IV monolayers with chair configuration to determine the energetically most stable state. The relative total energies of different magnetic states are shown in Table 3. All semihydrogenated group-IV monolayers with chair configuration except for SiC-H are ferromagnetic, indicated by the lowest total energy of FM. For SiC-H, the total energy of AFM is lower than the total energies of FM and NM by 10.6 and 251.6 meV, respectively, indicating that AFM coupling between silicon atoms is energetically the most favorable. For ferromagnetic materials, Curie temperature (*T*_*C*_) is a critical parameter at which ferromagnetic-paramagnetic transition occurs. According to the mean-field theory, Curie temperature of a ferromagnetic system can be estimated by the formula

**Table 3:** Relative total energies (per unit containing one hydrogen atom) of different magnetic states to the energetically most stable states of one-side semihydrogenated group-IV monolayers with chair configuration and Curie temperatures (*T*_*C*_) of ferromagnetic states. For each material (in each row), the lowest value among total energies of NM, FM, and AFM states is set to 0 meV

	NM (meV)	FM (meV)	AFM (meV)	*T*_*C*_ (K)
H-silicene	287.8	0	9.6	111.9
				121.6(c)
				300(d, e)
H-germanene	243.1	0	12.3	143.1
				144.8(c)
H-SiGe	251.3	0	8.1	93.4
				110(a)
SiGe-H	290.6	0	9.4	108.9
H-SiC	282.0	0	29.7	344.7
				340(b)
SiC-H	251.6	10.6	0	-
H-GeC	354.1	0	25.7	298.2
GeC-H	261.9	0	4.1	48.0

a, [45]; b, [48]; c [49]; d [50]; e [57]

$$ \gamma {K}_B{T}_C/2={E}_{\mathrm{AFM}}-{E}_{\mathrm{FM}} $$

where *γ* is the dimension of the system (*γ* = 2 in this work), *K*_*B*_ is the Boltzmann constant, and *E*_AFM_ and *E*_FM_ are the corresponding total energies for AFM and FM states, respectively [73, 74]. The Curie temperatures estimated by this formula are listed in Table 3. For SiGe-H, H-GeC, and GeC-H, it is the first time that their Curie temperatures are reported. By now, there is no experimental data of the Curie temperatures of semihydrogenated group-IV monolayers. For semihydrogenated silicene with chair configuration, our calculated Curie temperature is 111.9 K, comparable to 121.6 K calculated by Wang et al. [49] but much lower than 300 K calculated by Zhang et al. [50] and Zheng et al. [57]. For semihydrogenated germanene and monolayer SiC with chair configuration, our calculated Curie temperatures are in good agreement of the results reported by Xu et al. [48] and Wang et al. [49]. For H-SiGe, Zhou et al. [45] gave a Curie temperature of 110 K, slightly higher than our result of 93.4 K. Among these magnetic structures, H-GeC is a ferromagnetic semiconductor with a Curie temperature around room temperature.

**Fig. 8:**
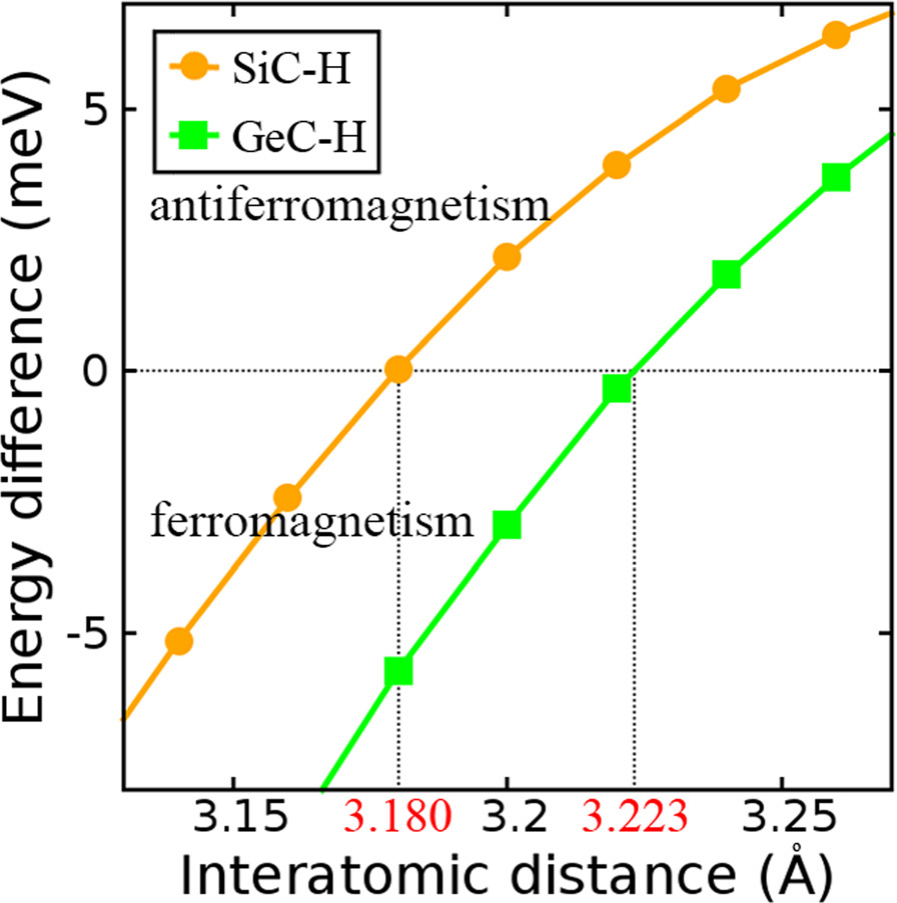
The difference of total energies between AFM and FM states for semihydrogenated monolayer SiC with chair SiC-H configuration (*yellow line*) and semihydrogenated monolayer GeC with chair GeC-H configuration (*green line*). The AFM-FM transition occurs at an interatomic distance of 3.180 Å (3.223 Å) for SiC-H (GeC-H)

The interatomic distance can considerably influence the exchange interaction between two neighboring magnetic atoms and then decides whether ferromagnetism or antiferromagnetism forms in magnetic materials. We test the effect of the interatomic distance to the magnetic coupling of semihydrogenated group-IV monolayers with chair configuration. For all structures with chair configuration in this work, the interatomic distance between two neighboring magnetic atoms is equal to the lattice constant. Total energies of FM and AFM coupling in each structure are recalculated with elongated and shorten lattice constants within a range of ±5 %. In this range, SiC-H and GeC-H undergo FM-AFM transitions. The difference of total energies between AFM and FM states is depicted in Fig. 8, where a positive value means a ferromagnetic system and a negative value means an antiferromagnetic system. The difference of total energies increases with elongated interatomic distance and decreases with shorten interatomic distance. FM-AFM transition for SiC-H and GeC-H occurs at 3.180 and 3.223 Å, respectively. This transition can prove that the interatomic distance between two neighboring magnetic atoms is a key factor that influences whether ferromagnetic or antiferromagnetic coupling forms. Kaloni et al. found that decorating silicene or germanene with 3d or 5d transition metal atoms can induce magnetism, and the formation of FM or AFM coupling is controlled by the species and position of transition metal atoms [33–36].

## Conclusions

Using first-principle calculations, structural, electronic, and magnetic properties of semihydrogenated and fully hydrogenated group-IV monolayers, including silicene, germanene, SiGe, SiC, and GeC, with boat, chair, and zigzag configurations are systematically studied. For semihydrogenated group-IV monolayers, zigzag configuration is found to have the lowest formation energy, while chair configuration is found to have the highest formation energy. For fully hydrogenated group-IV monolayers, energy of one-side hydrogenation is higher than that of both-side hydrogenation. Among boat, chair, and zigzag configurations of both-side full hydrogenation, chair configuration has the lowest energy.

Band structures of semihydrogenated germanene, SiGe, SiC, and GeC monolayers with boat and zigzag configurations are presented for the first time. Band gap opening due to semihydrogenation is predicted in silicene, germanene, and monolayer SiGe, regardless of the arrangement of hydrogen atoms, in contrast to reduced band gaps in semihydrogenated monolayer SiC and GeC. Semihydrogenated group-IV monolayers with boat and zigzag configurations are nonmagnetic, while semihydrogenated group-IV monolayers with chair configuration are magnetic. A two-step strategy is proposed to obtain magnetism in group-IV monolayers: first, fully hydrogenate the group-IV monolayers to get chair configuration on both side; second, remove hydrogen atoms from one side to get chair configuration of semihydrogenation on the other side. The spin moments are mainly carried by the group-IV atoms that are not bonded with hydrogen atoms. Semihydrogenated monolayer SiC with chair SiC-H configuration is an antiferromagnetic semiconductor, while other semihydrogenated group-IV monolayers with chair configuration are all ferromagnetic semiconductors. Our calculations indicate that the interatomic distance between two neighboring magnetic atoms can influence whether ferromagnetism or antiferromagnetism forms. If fully hydrogenated, semihydrogenated group-IV monolayers will become nonmagnetic semiconductors with larger band gaps. Our results will provide guidance for future researches of group-IV monolayer materials suitable for electronic and spintronic applications.

## Additional File

Additional file 1:
**(1) Lattice parameters and atomic positions of geometry-optimized one-side semihydrogenated group-IV monolayers with boat, chair, and zigzag configurations; (2) Lattice parameters and atomic positions of geometry-optimized one-side and both-side fully hydrogenated group-IV monolayers with boat, chair, and zigzag configurations; (3) Density of states of one-side semihydrogenated germanene, SiGe, and GeC monolayers with chair configuration; (4) Band structures of one-side and both-side fully hydrogenated group-IV monolayers with boat, chair, and zigzag configurations.** (DOCX 660 kb)
