# Supplementary material for: Band Gap Characters and Ferromagnetic/Antiferromagnetic Coupling in Group-IV Monolayers Tuned by Chemical Species and Hydrogen Adsorption Configurations
Source: Nanoscale Res Lett. 2015 Sep 4;10:351. doi: 10.1186/s11671-015-1040-y (PMC4559552; doi:10.1186/s11671-015-1040-y)
Supplement: Additional file 1: — (1) Lattice parameters and atomic positions of geometry-optimized one-side semihydrogenated group-IV monolayers with boat, chair, and zigzag configurations; (2) Lattice parameters and atomic positions of geometry-optimized one-side and both-side fully hydrogenated group-IV monolayers with boat, chair, and zigzag configurations; (3) Density of states of one-side semihydrogenated germanene, SiGe, and GeC monolayers with chair configuration; (4) Band structures of one-side and both-side fully hydrogenated group-IV monolayers with boat, chair, and zigzag configurations. (DOCX 660 kb) [file 11671_2015_1040_MOESM1_ESM.docx]

**Band gap characters and ferromagnetic/antiferromagnetic coupling in group-IV monolayers tuned by chemical species and hydrogen adsorption configurations**

Wen-Zhe Yu^†^, Jia-An Yan*^‡^, Shang-Peng Gao*^†^

†Department of Materials Science, Fudan University, Shanghai 200433, China

‡Department of Physics, Astronomy, and Geosciences, Towson University, Towson, MD 21252, USA

**Corresponding Authors**

*E-mail: jiaanyan@gmail.com (J.A. Yan), gaoshp@gmail.com (S.P. Gao).

**Supplementary Material**

**1. Lattice parameters and atomic positions of one-side semihydrogenated group-IV monolayers with boat, chair, and zigzag configurations**

**Semihydrogenated silicene**

**Chair**

Lattice parameters

a = b = 3.897 Å, c = 20 Å

Cell Angles

α = β = 90°, γ = 120°

Fractional coordinates of atoms

H 0.33333 0.66667 0.58488

Si_1_ 0.33333 0.66667 0.50932

Si_2_ 0.66667 0.33333 0.47581

**Boat**

Lattice parameters

a = 3.886 Å, b = 6.416 Å, c = 20 Å

Cell Angles

α = β = γ =90°

Fractional coordinates of atoms

H_1_ 0.00000 0.25015 0.41889

H_2_ 0.00000 0.71288 0.40850

Si_1_ 0.00000 0.30739 0.49171

Si_2_ 0.00000 0.67665 0.48318

Si_3_ 0.50000 0.18964 0.54730

Si_4_ 0.50000 0.86330 0.51042

**Zigzag**

Lattice parameters

a = 3.880 Å, b = 6.638 Å, c = 20 Å

Cell Angles

α = β = γ =90°

Fractional coordinates of atoms

H_1_ 0.00000 0.59431 0.41129

H_2_ 0.50000 0.91950 0.41332

Si_1_ 0.00000 0.32088 0.52096

Si_2_ 0.00000 0.65455 0.48403

Si_3_ 0.50000 0.15765 0.54534

Si_4_ 0.50000 0.85311 0.48507

**Semihydrogenated germanene**

**Chair**

Lattice parameters

a = b = 4.076 Å, c = 20 Å

Cell Angles

α = β = 90°, γ = 120°

Fractional coordinates of atoms

H 0.33333 0.66667 0.59156

Ge_1_ 0.33331 0.66669 0.51298

Ge_2_ 0.66670 0.33331 0.47546

**Boat**

Lattice parameters

a = 4.094 Å, b = 7.152 Å, c = 20 Å

Cell Angles

α = β = γ =90°

Fractional coordinates of atoms

H_1_ 0.00000 0.25778 0.41098

H_2_ 0.00000 0.68032 0.40485

Ge_1_ 0.00000 0.31451 0.48688

Ge_2_ 0.00000 0.67760 0.48348

Ge_3_ 0.50000 0.19107 0.54951

Ge_4_ 0.50000 0.87873 0.50430

**Zigzag**

Lattice parameters

a = 4.164 Å, b = 7.232 Å, c = 20 Å

Cell Angles

α = β = γ =90°

Fractional coordinates of atoms

H_1_ 0.00000 0.60015 0.40881

H_2_ 0.50000 0.91427 0.40828

Ge_1_ 0.00000 0.31810 0.50834

Ge_2_ 0.00000 0.65560 0.48513

Ge_3_ 0.50000 0.15883 0.54637

Ge_4_ 0.50000 0.85305 0.48307

**Semihydrogenated monolayer SiGe**

**Chair H-SiGe**

Lattice parameters

a = b = 4.003 Å, c = 20 Å

Cell Angles

α = β = 90°, γ = 120°

Fractional coordinates of atoms

H 0.33333 0.66667 0.58878

Si 0.33333 0.66667 0.51321

Ge 0.66667 0.33333 0.47801

**Chair SiGe-H**

Lattice parameters

a = b = 4.003 Å, c = 20 Å

Cell Angles

α = β = 90°, γ = 120°

Fractional coordinates of atoms

H 0.66667 0.33333 0.40931

Si 0.33333 0.66667 0.52289

Ge 0.66667 0.33333 0.48780

**Boat**

Lattice parameters

a = 4.008 Å, b = 6.997 Å, c = 20 Å

Cell Angles

α = β = γ =90°

Fractional coordinates of atoms

H_1_ 0.00000 0.69175 0.40746

H_2_ 0.00000 0.28374 0.41550

Si_1_  0.00000 0.31648 0.48967

Si_2_  0.50000 0.86215 0.50232

Ge_1_ 0.00000 0.67115 0.48581

Ge_2_ 0.50000 0.17474 0.53924

**Zigzag**

Lattice parameters

a = 4.046 Å, b = 7.041 Å, c = 20 Å

Cell Angles

α = β = γ =90°

Fractional coordinates of atoms

H_1_ 0.00000 0.60867 0.40737

H_2_ 0.50000 0.90720 0.41184

Si_1_  0.00000 0.31776 0.51083

Si_2_  0.50000 0.84986 0.48413

Ge_1_ 0.00000 0.65605 0.48414

Ge_2_ 0.50000 0.16047 0.54168

**Semihydrogenated monolayer SiC**

**Chair H-SiC**

Lattice parameters

a = b = 3.141 Å, c = 20 Å

Cell Angles

α = β = 90°, γ = 120°

Fractional coordinates of atoms

H 0.66670 0.33330 0.57373

C 0.33331 0.66669 0.47869

Si 0.66665 0.33335 0.49759

**Chair SiC-H**

Lattice parameters

a = b = 3.100 Å, c = 20 Å

Cell Angles

α = β = 90°, γ = 120°

Fractional coordinates of atoms

H 0.33333 0.66667 0.56357

C 0.33333 0.66667 0.50779

Si 0.66667 0.33333 0.47864

**Boat**

Lattice parameters

a = 3.150 Å, b = 5.492 Å, c = 20 Å

Cell Angles

α = β = γ =90°

Fractional coordinates of atoms

H_1_  0.00000 0.29442 0.44759

H_2_  0.00000 0.69195 0.41779

C_1_  0.00000 0.33204 0.50271

C_2_  0.50000 0.83845 0.51932

Si_1_ 0.00000 0.68822 0.49332

Si_2_ 0.50000 0.15492 0.51927

**Zigzag**

Lattice parameters

a = 3.170 Å, b = 5.492 Å, c = 20 Å

Cell Angles

α = β = γ =90°

Fractional coordinates of atoms

H_1_  0.00000 0.60570 0.41596

H_2_  0.50000 0.89885 0.44526

C_1_  0.00000 0.33084 0.52503

C_2_  0.50000 0.84269 0.49910

Si_1_ 0.00000 0.64850 0.49025

Si_2_ 0.50000 0.17343 0.52441

**Semihydrogenated monolayer GeC**

**Chair H-GeC**

Lattice parameters

a = b = 3.288 Å, c = 20 Å

Cell Angles

α = β = 90°, γ = 120°

Fractional coordinates of atoms

H 0.33333 0.66667 0.58616

C 0.66667 0.33333 0.48637

Ge 0.33333 0.66667 0.50747

**Chair GeC-H**

Lattice parameters

a = b = 3.291 Å, c = 20 Å

Cell Angles

α = β = 90°, γ = 120°

Fractional coordinates of atoms

H 0.66667 0.33333 0.56397

C 0.66667 0.33333 0.50851

Ge 0.33333 0.66667 0.47753

**Boat**

Lattice parameters

a = 3.300 Å, b = 5.675 Å, c = 20 Å

Cell Angles

α = β = γ =90°

Fractional coordinates of atoms

H_1_ 0.00000 0.70993 0.44194

H_2_ 0.00000 0.30277 0.40965

C_1_ 0.00000 0.66848 0.49637

C_2_ 0.50000 0.16339 0.51665

Ge_1_ 0.50000 0.84340 0.51735

Ge_2_ 0.00000 0.31204 0.48804

**Zigzag**

Lattice parameters

a = 3.332 Å, b = 5.780 Å, c = 20 Å

Cell Angles

α = β = γ =90°

Fractional coordinates of atoms

H_1_ 0.00000 0.60523 0.43926

H_2_ 0.50000 0.89469 0.40872

C_1_ 0.00000 0.65603 0.49290

C_2_ 0.50000 0.16822 0.52195

Ge_1_ 0.00000 0.32595 0.52147

Ge_2_ 0.50000 0.84988 0.48571

**2. Lattice parameters and atomic positions of one-side and both-side fully hydrogenated group-IV monolayers with boat, chair, and zigzag configurations.**

**Fully hydrogenated silicene**

**One-side**

Lattice parameters

a = b = 4.138 Å, c = 20 Å

Cell Angles

α = β = 90°, γ = 120°

Fractional coordinates of atoms

H_1_ 0.33333 0.66667 0.57746

H_2_ 0.66667 0.33333 0.57746

Si_1_ 0.33333 0.66667 0.50255

Si_2_ 0.66667 0.33333 0.50255

**Both-side chair**

Lattice parameters

a = b = 3.891 Å, c = 20 Å

Cell Angles

α = β = 90°, γ = 120°

Fractional coordinates of atoms

H_1_ 0.33333 0.66667 0.40682

H_2_ 0.66667 0.33333 0.59318

Si_1_ 0.33333 0.66667 0.48194

Si_2_ 0.66667 0.33333 0.51806

**Both-side boat**

Lattice parameters

a = 3.865 Å, b = 6.474 Å, c = 20 Å

Cell Angles

α = β = γ =90°

Fractional coordinates of atoms

H_1_ 0.00000 0.24403 0.40270

H_2_ 0.00000 0.75597 0.40270

H_3_ 0.50000 0.25597 0.59730

H_4_ 0.50000 0.74403 0.59730

Si_1_ 0.00000 0.31667 0.47409

Si_2_ 0.00000 0.68333 0.47409

Si_3_ 0.50000 0.18333 0.52591

Si_4_ 0.50000 0.81667 0.52591

**Both-side zigzag**

Lattice parameters

a = 3.888 Å, b = 6.004 Å, c = 20 Å

Cell Angles

α = β = γ =90°

Fractional coordinates of atoms

H_1_ 0.00000 0.47632 0.60858

H_2_ 0.00000 0.52368 0.39142

H_3_ 0.50000 0.02368 0.60858

H_4_ 0.50000 0.97632 0.39142

Si_1_ 0.00000 0.36177 0.54174

Si_2_ 0.00000 0.63823 0.45826

Si_3_ 0.50000 0.13823 0.54174

Si_4_ 0.50000 0.86177 0.45826

**Fully hydrogenated germanene**

**One-side**

Lattice parameters

a = b = 4.340 Å, c = 20 Å

Cell Angles

α = β = 90°, γ = 120°

Fractional coordinates of atoms

H_1_ 0.33333 0.66667 0.57911

H_2_ 0.66667 0.33333 0.57911

Ge_1_ 0.33333 0.66667 0.50089

Ge_2_ 0.66667 0.33333 0.50089

**Both-side chair**

Lattice parameters

a = b = 4.080 Å, c = 20 Å

Cell Angles

α = β = 90°, γ = 120°

Fractional coordinates of atoms

H_1_ 0.33333 0.66667 0.40334

H_2_ 0.66667 0.33333 0.59666

Ge_1_ 0.33333 0.66667 0.48128

Ge_2_ 0.66667 0.33333 0.51872

**Both-side boat**

Lattice parameters

a = 4.059 Å, b = 6.872 Å, c = 20 Å

Cell Angles

α = β = γ = 90°

Fractional coordinates of atoms

H_1_ 0.00000 0.26131 0.39887

H_2_ 0.00000 0.73869 0.39887

H_3_ 0.50000 0.23869 0.60113

H_4_ 0.50000 0.76131 0.60113

Ge_1_ 0.00000 0.31926 0.47427

Ge_2_ 0.00000 0.68074 0.47427

Ge_3_ 0.50000 0.18074 0.52573

Ge_4_ 0.50000 0.81926 0.52573

**Both-side zigzag**

Lattice parameters

a = 4.106 Å, b = 6.666 Å, c = 20 Å

Cell Angles

α = β = γ = 90°

Fractional coordinates of atoms

H_1_ 0.00000 0.43326 0.61071

H_2_ 0.00000 0.56674 0.38929

H_3_ 0.50000 0.06674 0.61071

H_4_ 0.50000 0.93326 0.38929

Ge_1_ 0.00000 0.35393 0.53731

Ge_2_ 0.00000 0.64607 0.46269

Ge_3_ 0.50000 0.14607 0.53731

Ge_4_ 0.50000 0.85393 0.46269

**Fully hydrogenated monolayer SiGe**

**One-side**

Lattice parameters

a = b = 4.234 Å, c = 20 Å

Cell Angles

α = β = 90°, γ = 120°

Fractional coordinates of atoms

H_1_ 0.33333 0.66667 0.57885

H_2_ 0.66667 0.33333 0.57770

Si 0.66667 0.33333 0.50288

Ge 0.33333 0.66667 0.50057

**Both-side chair**

Lattice parameters

a = b = 3.986 Å, c = 20 Å

Cell Angles

α = β = 90°, γ = 120°

Fractional coordinates of atoms

H_1_ 0.33333 0.66667 0.59410

H_2_ 0.66667 0.33333 0.40448

Si 0.33333 0.66667 0.51903

Ge 0.66667 0.33333 0.48239

**Both-side boat**

Lattice parameters

a = 3.961 Å, b = 6.612 Å, c = 20 Å

Cell Angles

α = β = γ =90°

Fractional coordinates of atoms

H_1_ 0.00000 0.25520 0.40187

H_2_ 0.00000 0.74283 0.39974

H_3_ 0.50000 0.24283 0.60026

H_4_ 0.50000 0.75520 0.59813

Si_1_  0.00000 0.18456 0.47327

Si_2_  0.50000 0.68456 0.52673

Ge_1_ 0.00000 0.31742 0.52627

Ge_2_ 0.50000 0.81742 0.47373

**Both-side zigzag**

Lattice parameters

a = 3.984 Å, b = 6.101 Å, c = 20 Å

Cell Angles

α = β = γ =90°

Fractional coordinates of atoms

H_1_ 0.00000 0.02271 0.60969

H_2_ 0.00000 0.97934 0.38801

H_3_ 0.50000 0.47934 0.61199

H_4_ 0.50000 0.52271 0.39031

Si_1_  0.00000 0.13682 0.54314

Si_2_  0.50000 0.63682 0.45686

Ge_1_ 0.00000 0.86112 0.45722

Ge_2_ 0.50000 0.36112 0.54278

**Fully hydrogenated monolayer SiC**

**One-side**

Lattice parameters

a = b = 3.269 Å, c = 20 Å

Cell Angles

α = β = 90°, γ = 120°

Fractional coordinates of atoms

H_1_  0.33333 0.66667 0.58898

H_2_  0.66667 0.33333 0.55698

C 0.66667 0.33333 0.50112

Si 0.33333 0.66667 0.51292

**Both-side chair**

Lattice parameters

a = b = 3.121 Å, c = 20 Å

Cell Angles

α = β = 90°, γ = 120°

Fractional coordinates of atoms

H_1_  0.33333 0.66667 0.57464

H_2_  0.66667 0.33333 0.41559

C 0.33333 0.66667 0.51928

Si 0.66667 0.33333 0.49049

**Both-side boat**

Lattice parameters

a = 3.111 Å, b = 5.223 Å, c = 20 Å

Cell Angles

α = β = γ =90°

Fractional coordinates of atoms

H_1_  0.00000 0.24858 0.42805

H_2_  0.00000 0.74290 0.40705

H_3_  0.50000 0.24290 0.59295

H_4_  0.50000 0.74858 0.57195

C_1_  0.00000 0.18620 0.48086

C_2_  0.50000 0.68620 0.51914

Si_1_ 0.00000 0.82232 0.47923

Si_2_ 0.50000 0.32232 0.52077

**Both-side zigzag**

Lattice parameters

a = 3.122 Å, b = 4.630 Å, c = 20 Å

Cell Angles

α = β = γ =90°

Fractional coordinates of atoms

H_1_  0.00000 0.01822 0.39836

H_2_  0.00000 0.99990 0.58099

H_3_  0.50000 0.49990 0.41901

H_4_  0.50000 0.51822 0.60164

C_1_  0.00000 0.12633 0.53404

C_2_  0.50000 0.62633 0.46596

Si_1_ 0.00000 0.85556 0.46345

Si_2_ 0.50000 0.35556 0.53655

**Fully hydrogenated monolayer GeC**

**One-side**

Lattice parameters

a = b = 3.475 Å, c = 20 Å

Cell Angles

α = β = 90°, γ = 120°

Fractional coordinates of atoms

H_1_ 0.33333 0.66667 0.58985

H_2_ 0.66667 0.33333 0.55729

C 0.66667 0.33333 0.50211

Ge 0.33333 0.66667 0.51074

**Both-side chair**

Lattice parameters

a = b = 3.273 Å, c = 20 Å

Cell Angles

α = β = 90°, γ = 120°

Fractional coordinates of atoms

H_1_ 0.33333 0.66667 0.57610

H_2_ 0.66667 0.33333 0.41257

C 0.33333 0.66667 0.52098

Ge 0.66667 0.33333 0.49034

**Both-side boat**

Lattice parameters

a = 3.259 Å, b = 5.467 Å, c = 20 Å

Cell Angles

α = β = γ =90°

Fractional coordinates of atoms

H_1_ 0.00000 0.24834 0.42672

H_2_ 0.00000 0.74032 0.40321

H_3_ 0.50000 0.24032 0.59679

H_4_ 0.50000 0.74834 0.57328

C_1_ 0.00000 0.18820 0.47928

C_2_ 0.50000 0.68820 0.52072

Ge_1_ 0.00000 0.82314 0.47787

Ge_2_ 0.50000 0.32314 0.52213

**Both-side zigzag**

Lattice parameters

a = 3.277 Å, b = 5.075 Å, c = 20 Å

Cell Angles

α = β = γ =90°

Fractional coordinates of atoms

H_1_ 0.00000 0.03270 0.58161

H_2_ 0.00000 0.98098 0.39282

H_3_ 0.50000 0.48098 0.60718

H_4_ 0.50000 0.53270 0.41839

C_1_ 0.00000 0.13258 0.53267

C_2_ 0.50000 0.63258 0.46733

Ge_1_ 0.00000 0.85374 0.46379

Ge_2_ 0.50000 0.35374 0.53621

**3. Density of states of one-side semihydrogenated germanene, SiGe, and GeC monolayers with chair configuration.**


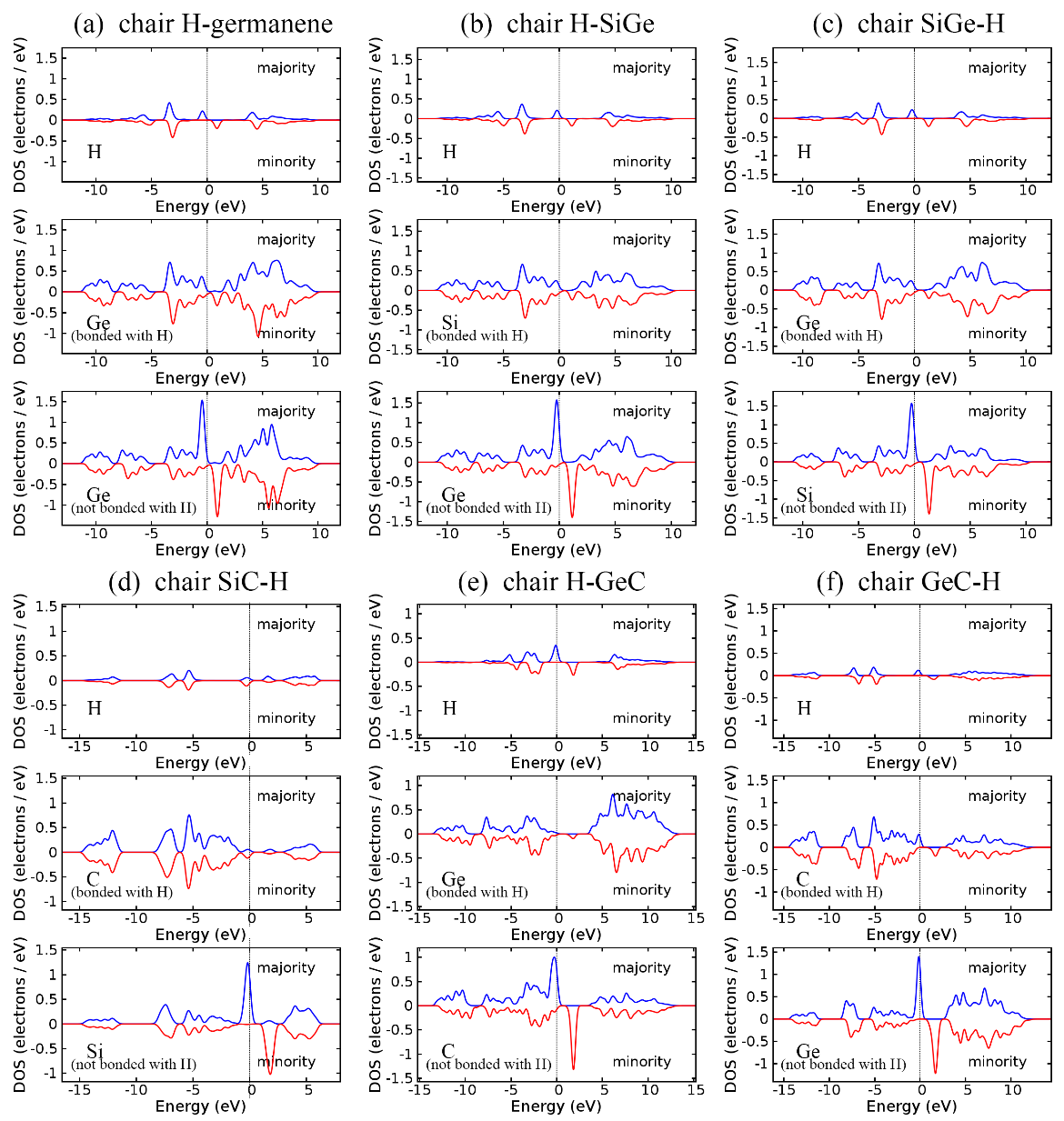


**Figure S1.** Density of states (DOS) located at each atom in semihydrogenated germanene (a), SiGe (b, c), SiC (d), and GeC (e, f) monolayers with chair configuration.

**4. Band structures of one-side and both-side fully hydrogenated group-IV monolayers with boat, chair, and zigzag configurations.**


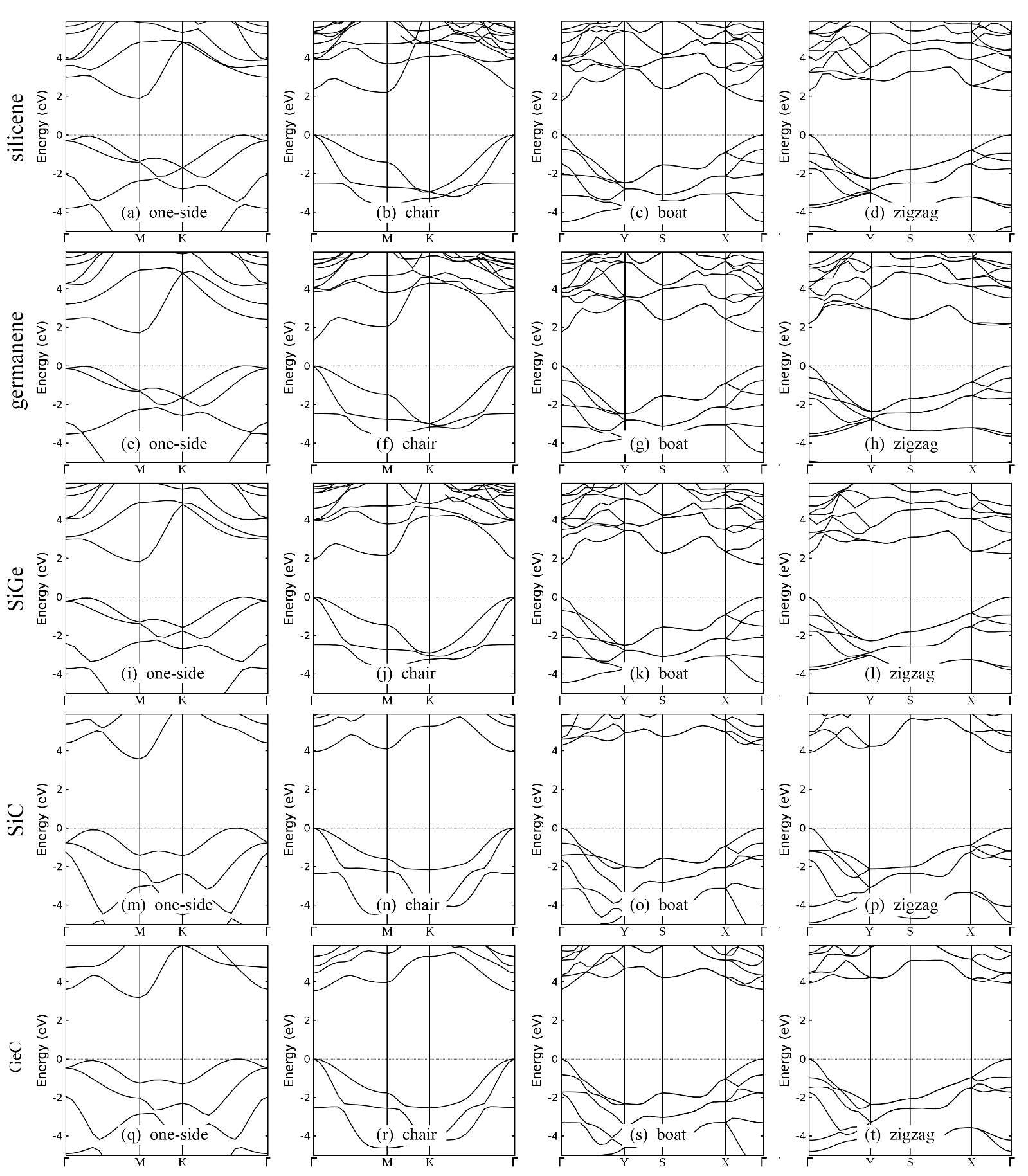


**Figure S2.** Band structures of one-side and both-side fully hydrogenated silicene, germanene, SiGe, SiC, and GeC monolayers with chair, boat, and zigzag configurations. The valence band maximum has been set to 0 eV and indicated by the black dotted line.
